# Intrafamilial Transmission of Pneumococcal Acute Spontaneous Peritonitis

**DOI:** 10.3390/idr15030030

**Published:** 2023-05-30

**Authors:** Ioanna Papadatou, Angeliki Moudaki, Anastasia Mentessidou, Dimitrios Tsakogiannis, Elissavet Georgiadou, Vana Spoulou

**Affiliations:** 1 First Department of Paediatrics, National and Kapodistrian University of Athens, ‘Aghia Sophia’ Children’s Hospital, 115 27 Athens, Greece; 2 Immunology and Vaccinology Research Lab, First Department of Paediatrics, National and Kapodistrian University of Athens, 115 27 Athens, Greece; 3 University Research Institute for the Study of Genetic & Malignant Disorders in Childhood, School of Medicine, National and Kapodistrian University of Athens, ‘Aghia Sophia’ Children’s Hospital, 115 27 Athens, Greece; 4 Department of Paediatric Surgery, ‘Aghia Sophia’ Children’s Hospital, 115 27 Athens, Greece; 5 Department of Microbiology, Athens Medical School, 115 27 Athens, Greece

**Keywords:** pneumococcal peritonitis, pneumococcal serotype 1, invasive pneumococcal disease

## Abstract

*Streptococcus pneumonia* (*S. pneumoniae*, Pneumococcus) is a major cause of childhood morbidity and mortality worldwide. The most common presentations of invasive pneumococcal disease (IPD) in children include bacteremic pneumonia, meningitis, and septicemia. However, pneumococcal acute spontaneous peritonitis is a highly uncommon—and potentially life-threatening—presentation of invasive pneumococcal disease and should be considered in cases of abdominal sepsis. To our knowledge, we report the first case of intrafamilial transmission of pneumococcal peritonitis in two previously healthy children.

## 1. Introduction

*Streptococcus pneumonia* (*S. pneumoniae*, Pneumococcus) is a major cause of childhood morbidity and mortality worldwide. The most common presentations of invasive pneumococcal disease (IPD) in children include bacteremic pneumonia, meningitis, and septicemia [1]. Pneumococcal peritonitis is an uncommon presentation of IPD, representing 1% of IPD cases.

Spontaneous bacterial peritonitis (SBP) is defined as a diffuse infective inflammation of the peritoneal cavity without an evident or identifiable source of infection [2]. Gram-negative pathogens account for the majority of SBP cases (approximately 75%), with *E. coli*, *Klebsiella pneumonia*, and *Pseudomonas* spp. being the most isolated microorganisms. Gram-positive aerobic organisms, mainly *S. pneumoniae* and Viridans group streptococci, are responsible for the remaining cases, while anaerobic bacteria are found in less than 5% of cases [2].

In Europe, the implementation of universal infant immunization with pneumococcal conjugate vaccines (PCVs) in the early 2000s has significantly reduced the burden of invasive pneumococcal disease (IPD) among children of all ages [3]. However, small communities that refuse immunizations for cultural and religious reasons are scattered across Southern and Northern Europe and together with incoming refugees represent significant pouches of population susceptible to vaccine-preventable diseases.

Pneumococcal SBP is a potentially life-threatening condition that is highly uncommon in healthy children. A rise in disease incidence over the last decade has been documented [4], with recent reports from Latin America estimating that pneumococcal SBP accounts for up to 8–10% of abdominal emergencies in children [5]. However, reports of pneumococcal SBP in otherwise healthy children have been scarce [5]. To our knowledge, this is the first report on intrafamilial transmission of pneumococcal SBP in previously healthy children.

## 2. Case Presentation

A 3-year-old girl and 7-year-old girl were brought to the pediatric emergency department due to a 7-day history of cough, 2-day history of high fever (up to 40 °C), and diarrhea (approx. 10/day). The two girls are cousins and live in the same household. 

Both children presented as critically unwell. In detail, the 3-year-old girl (hereafter referred to as Patient 1) was lethargic and responsive only to pain, with signs of severe dehydration, tachycardia, and tachypnea. Upon chest auscultation, reduced breathing sounds in the left lower lobe were found, while examination of the abdomen revealed tenderness, generalized abdominal wall guarding, and mild abdominal distension. Bowel sounds were scarce. 

The 7-year-old (hereafter referred to as Patient 2) was lethargic and responsive to voice, with signs of mild dehydration, tachycardia, and tachypnea. Breathing sounds were normal and examination of the abdomen revealed significant abdominal distension, tenderness, and generalized abdominal wall guarding and absent bowel sounds.

Past medical history of both children was unremarkable, but inconsistent medical follow-up and incomplete immunizations were reported. According to parents, both patients had received a single dose of the 13-valent pneumococcal vaccine during the first year of their lives. Documentation of immunization records was not presented.

A full septic screen and chest and abdominal imaging were performed in both children. Patient 1 had leukocytopenia with a white blood cell count of 3.860/μL (84% neutrophils, 8% lymphocytes, 3% monocytes), C-reactive protein levels of 475 mg/L and procalcitonin levels of 26.12 ng/mL. Urea and electrolytes were within the normal range except mild hyponatremia (Na 130 mmol/L). Patient 2 had leukocytosis with a white blood cell count of 21,690/μL (95.6% neutrophils, 2.2% lymphocytes, 0.8% monocytes) and a C-reactive protein level of 321 mg/L. Urea and electrolytes were within the normal range except mild hyponatremia (Na 129 mmol/L) (Table 1). 

Chest X-rays showed an opacity in the left lower lobe in Patient 1 and normal findings in Patient 2. Abdominal X-rays were unremarkable. Abdominal ultrasounds revealed generalized intestinal wall thickening and free intraperitoneal fluid, while the appendix was not visualized in either patient.

**Figure 1:**
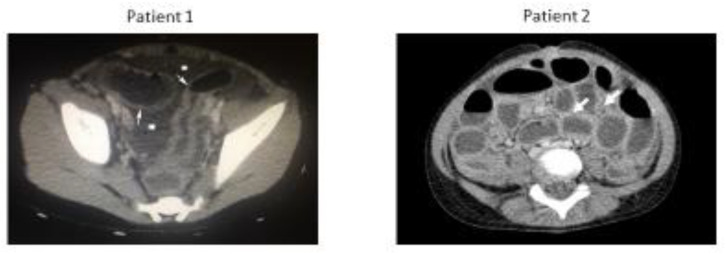
Contrast-enhanced transverse computed tomography imaging view of the abdomen of Patients 1 and 2. Free intraperitoneal fluid (asterisks) and intestinal wall thickening (arrows) can be seen.

Patients were initially diagnosed as having cases of generalized bacterial enteritis with sepsis and empirical intravenous antibiotic therapy was initiated with Cefotaxime, Amikacin, and Metronidazole. Due to accumulating clinical signs of peritonitis and inconclusive ultrasound findings, computed tomography (CT) was performed at approximately 12 h post-admission in both patients. CT images confirmed the presence of generalized intestinal wall thickening and free intraperitoneal fluid in both children, but we could not rule out that these findings were secondary to appendiceal rupture (Figure 1).

Emergency diagnostic laparotomy was performed in both children due to persistent signs of peritonitis at 24 and 48 h post-admission in Patients 2 and 1, respectively. Intraoperatively, free purulent fluid and diffuse fibrin deposition in the abdomen and pelvis were found, findings indicative of diffuse inflammation of the peritoneum. No underlying cause was found, as the viscera, including the appendix, were macroscopically normal. The appendix was removed in both patients, and the absence of appendiceal rupture was microscopically confirmed.

Blood culture and multiple PCR (BioFire, Biomerieux, Paris, France) as well as intrabdominal fibrinous tissue culture showed *S. pneumoniae*. Additional pneumococcal serotype identification by means of capsular sequence typing (CST) was performed in the National Meningitis Reference Laboratory (NMRL), and pneumococcal serotype 1 was detected. The diagnosis of acute spontaneous pneumococcal peritonitis and septicemia was made for both children. Consequently, sequencing of the Pneumococcal Surface Protein A (PspA) from the two strains isolated was performed. Briefly, a partial region of the PspA gene (approximately 1200 bp in size) was amplified through PCR using the primer set LSM12/SKH2. Amplicons were sequenced at EurofinsGenomics/VBC Biotech, Vienna, Austria. The sequences were characterized using database alignments at the National Centre for Biotechnology Information (NCBI) website and the mega blast algorithm (http://blast.ncbi.nlm.nih.gov/Blast.cgi, accessed on 24 March 2019). The % nucleotide and amino acid sequence similarity of the two isolated bacterial strains was determined using MolSoft ICM 2.7 program. Nucleotide and amino acid sequence alignment of the two isolated strains revealed 100% sequence similarity, providing evidence for intrafamilial transmission of pneumococcal SBP (Appendix A, Table A1).

Antigen-specific antibodies against the invading serotype 1 and three additional vaccine serotypes (9V, 3, and 19A) at one week post-presentation were measured using ELISA. Both patients had high protective antibody titers (>0.35 μg/mL) against vaccine serotypes 9V, 19A, and 3, confirming the reported immunization history with PCV13 and reflecting normal humoral response to immunization. At one week post-presentation, antibodies against the invading serotype 1 were also high, suggesting that pre-existing vaccine-induced antibodies against serotype 1 might not have been sufficient to protect against IPD at the time of infection, but the host immune response to natural infection was not defective (Table 2).

Post-operatively, the two girls completed a 14-day course of intravenous antibiotics and showed progressive uneventful recovery. A follow-up was arranged in the Infectious Diseases and Immunology clinic at one-month post-discharge, but the family did not present.

Two years later, Patient 1 presented to our clinic with minor flu-like symptoms. Upon this opportunity, we rearranged immunological screening for both patients. Total immunoglobulins G, A, and M, complement factors C3 and C4, and immunophenotyping of peripheral blood NK, T, and B lymphocytes via flow cytometry were within the normal range for both children according to their age (Table 3).

## 3. Discussion

Spontaneous bacterial peritonitis (SBP) in children caused by *S. pneumoniae* has been recognized for more than a century, and it was first described by Bozzolo et al. in 1885 [4]. Pneumococcal SBP is highly uncommon in healthy children, and affects mainly patients with nephrotic syndrome, liver cirrhosis, and HIV infection [3,4], with a peak incidence between 4 and 9 years of age [4,5]. The most common clinical manifestations of pneumococcal SBP are abdominal pain, high fever, diarrhea, and vomiting [3,4].

A rise in disease incidence over the last decade has been documented [4], with recent reports from Latin America estimating that pneumococcal SBP accounts for up to 8–10% of abdominal emergencies in children [7]. However, reports of pneumococcal SBP in otherwise healthy children have been scarce [7]. To our knowledge, this is the first report on intrafamilial transmission of pneumococcal SBP in previously healthy children.

The pathogenesis of pneumococcal peritonitis remains unclear. Pneumococci can either enter the peritoneal cavity via the genital tract in young women or hematogenously following a respiratory tract infection [3,4,5,6,7]. In our case, we postulate that the pathogen was initially transmitted through the respiratory track, causing left lower lobe consolidation in Patient 1, and consequently spread to the peritoneum hematogenously. For Patient 2, the route of infection is less clear, but we postulate transmission through the respiratory track by the infectious Patient 1 via droplets and then hematogenous spread to the peritoneum.

The association of serotype 1 with primary peritonitis has also been reported by others [5,6,7]. In a pediatric case series from South America [7], 79.7% of the 64 identified serotypes causing peritonitis were serotype 1. Moreover, serotype 1 seems to have unique characteristics compared to other pneumococcal serotypes [8]: firstly, IPD cases caused by serotype 1 have significantly higher proportions of bacteremic pneumonia (with or without pleural effusion) and spontaneous peritonitis. Moreover, serotype 1 IPD affected older children more often than other common pneumococcal serotypes and caused IPD in healthy children more frequently that in children with underlying diseases in Israel [8].

Interestingly, in our case, not one but two members of the same household acquired serotype 1 invasive disease. Serotype 1 is known to have a low carriage rate and high disease potential and thus a high case-to-carrier ratio. It has also been associated with ongoing outbreaks of pneumococcal meningitis among older children and adults in the African meningitis belt despite the implementation of an infant PCV13 vaccination schedule [9].

The interfamilial transmission of serotype 1 pneumococcal SBP seen in this case could be attributed either to the increased invasive potential of the specific strain with tropism to the peritoneal cavity or to an unknown genetic factor of host susceptibility to pneumococcal peritonitis. As seen in other presentations of IPD before [10], genetic variations in innate and adaptive immune genes shared between family members could alter the immune response and influence susceptibility to pneumococcal infection. Serological studies demonstrate an adequate humoral immune response to pneumococcal vaccine antigens and a high antibody response against the invading serotype following IPD.

## 4. Conclusions

Spontaneous pneumococcal peritonitis is a rare but potentially life-threatening IPD manifestation and should be considered in cases of abdominal sepsis. Clinicians should include spontaneous pneumococcal peritonitis in the differential diagnosis of abdominal sepsis and include antibiotic coverage for Gram-positive bacteria in the empiric therapy of abdominal sepsis until microbiology testing comes through. In contrast with the much more common surgical causes of abdominal sepsis, intrafamilial transmission is possible in spontaneous pneumococcal peritonitis and should give rise to high suspicion for the disease.

## Figures and Tables

**Table 1:** Laboratory Values and Clinical Findings Upon Hospital Admission for Patients 1 and 2.

Laboratory Values Upon Hospital Admission		Patient 1	Patient 2
**WBC**	×10³/μL	3.8	21.69
**Neutrophils**	%	84	95.6
**Lymphocytes**	%	8	2.2
**Monocytes**	%	3	0.8
**Hemoglobulin (HGb)**	g/dL	**9.4**	11.5
**Hematocrit (HCT)**	%	**29.5**	34.2
**Platelets (PLTs)**	×10³/μL	343	241
**C-reactive protein**	mg/L	**475**	**321**
**Procalcitonin (PCT)**	ng/mL	**26.12**	
**Glucose**	mg/dL	109	84
**Urea**	mg/dL	10	29
**Creatinine**	mg/dL	0.25	0.40
**SGOT(AST)**	U/L	43	13
**SGPT(ALT)**	U/L	6	6
**γ-GT**	U/L	7	10
**ALP**	U/L	66	140
**Total Billirubin**	mg/dL	0.26	0.18
**Direct Billirubin**	mg/dL	0.12	0.09
**Total Protein**	g/dL	5.2	7.2
**Albumin**	g/dL	3.2	4.0
**Potassium**	mmol/L	3.6	3.9
**Sodium**	mmol/L	**130**	**129**
**Calcium**	mg/dL	8.6	10.1
**Signs and Symptoms Upon Hospital Admission**		**Patient 1**	**Patient 2**
**Level of**		yes	yes
**Tachypnoea & Tachycardia**		yes	yes
**Dehydration (by clinical findings)**		severe	moderate
**Auscultation of the Chest (findings)**		left lower lobe: reduced breathing sounds	No abnormality detected (NAD)
**Palpitation of the Abdomen (findings)**		Tenderness; generalized abdominal wall guarding; mild abdominal distension	significant abdominal distension;tenderness; generalized abdominal wall guarding
**Auscultation of the Abdomen (findings)**		Scarce bowel sounds	Absent bowel sounds

Abnormal values in bold.

**Table 2:** Serum antibody concentration against the invading serotype 1 and three additional serotypes, which are included in the 13-valent pneumococcal conjugated vaccine.

Patient	Protective Antibody Levels against IPD [6]	Serotype 1	Serotype 3	Serotype 9V	Serotype 19A
1 (3-year-old)	>0.5 mg/L	5.08 mg/L	0.41 mg/L	16.21 mg/L	20.32 mg/L
2 (7-year-old)	>0.5 mg/L	7.32 mg/L	0.85 mg/L	8.01 mg/L	11.73 mg/L

**Table 3:** Immunology work-up of Patients 1 and 2.

Immunoglobulins and Complement Factors		Patient 1 (Normal Range for Age)	Patient 2 (Normal Range for Age)
IgG (nephelometry)	mg/dL	2090.00 (955–1995)	1810.00 (891–2042)
IgA (nephelometry)	mg/dL	185 (85–214)	163 (52–331)
IgM (nephelometry)	mg/dL	148 (65–372)	139 (63–275)
C3 (nephelometry)	mg/dL	196 (90–180)	147 (90–180)
C4 (nephelometry)	mg/dL	57 (10–40)	30 (10–40)

## Data Availability

The whole sequencing data are provided in the appendix. A link is provided in cases where the sequencing is not given in full within the submission.

## Appendix A

**Table A1:** Sequencing of the PspA gene and protein of the two pneumococcal strains, isolated from the 3-year-old and the 7-year-old girl, revealed 100% alignment, providing evidence of intrafamilial transmission of pneumococcal spontaneous peritonitis in our patients.

**Patient 1 (3-Year-Old) PspA Gene**
AGCCTACTGTTGTAAGAGCAGAAGAAGCCCCCGTAGCTAGTCAGTCTAAAGCTGAAAAAGACTATGATGCAGCGAAGAGAGACGCTGAGAATGCGAAAAAAGCTTTAGAAGAAGCAAAACGTGCGCAGAAAAAATATGAGGATGATCAGAAGAAAACTGAGGAGAAAGCGAAAAAAGAGAAAGAAGCTTCTAAAGAGGAACAAGCTGCAAATCTGAAATATCAACAAGAGTTGGTTAAATATGCTAGTGAAAAAGATTCAGTAAAAAAAGCTAAAATTCTGAAAGAAGTGGAGGAAGCTGAGAAAGAGCATAAGAAAAAACGAGCAGAATTTGAGAAAGTTAGATCAGAGGTAATTCCTAGCGCGGAAGAGTTAAAAAAGACTAGACAAAAAGCAGAAGAGGCTAAAGCAAAAGAAGCAGAACTTATTAAAAAAGTAGAAGAAGCTGAGAAAAAAGTTACTGAAGCCAAACAAAAATTGGATGCTGAACGTGCTAAAGAAGTTGCTCTTCAAGCCAAAATCGCTGAGTTGGAAAATGAAGTTTATAGACTAGAAACAGAACTCAAAGGGATTGATGAATCTGACTCAGAAGATTATGTTAAAGAAGGTCTCCGTGCTCCTCTTCAATCTGAATTGGATGCCAAACGAACTAAACTATCAACACTTGAAGAGTTGAGTGATAAGATTGATGAGTTAGACGCTGAAATTGCAAAACTTGAAAAAAATGTAGAATATTTCAAAAAAACCGATGCTGAGCAAACTGAACAATACCTTGCTGCAGCTGAAAAAGACTTAGCTGATAAAAAAGCTGAATTGGAGAAAACTGAAGCTGACCTTAAGAAAGCAGTTAATGAGCCAGAAAAACCAGCTGAAGAAACTCCAGCTCCAGCACCAAAACCAGAAAAAACAGATGATCAACAAGCTGAAGAAGACTATGCTCGTAGATCAGAAGAAGAATATAACCGCTTGCCCCAACAGCAACCGCCAAAAGCAGAAAAACCAGCTCCAGCACCAAAACCAGAGCAACCAGTTCCT
**Patient 2 (7-Year-Old) PspA Gene**
AGCCTACTGTTGTAAGAGCAGAAGAAGCCCCCGTAGCTAGTCAGTCTAAAGCTGAAAAAGACTATGATGCAGCGAAGAGAGACGCTGAGAATGCGAAAAAAGCTTTAGAAGAAGCAAAACGTGCGCaGAAAAAATATGAGGATGATCAGAAGAAAACTGAGGAGAAAGCGAAAAAAGAGAAAGAAGCTTCTAAAGAGGAACAAGCTGCAAATCTGAAATATCAaCAAGAGTTGGTTAAATATGCTAGTGAAAAAGATTCAGTAAAAaAAGCTAAAATTCTGAAAGAAGTGGAGGAAGCTGAGAAAGAGCATAAGAAAAAACGAGCAGAATTTGAGAAAGTTAGATCAGAGGTAATTCCTAGCGCGGAAGAGTTAAAAAAGACTAGACAAAAAGCAGAAGAGGCTAAAGCAAAAGAAGCAGAACTTATTAAAAAAGTAGAAGAAGCTGAGAAAAAAGTTACTGAAGCCAAACAAAAATTGGATGCTGAACGTGCTAAAGAAGTTGCTCTTCAAGCCAAAATCGCTGAGTTGGAAAATGAAGTTTATAGACTAGAAACAGAACTCAAAGGGATTGATGAATCTGACTCAgAAGATTATGTTAAAGAAGGTCTCCGTGCTCCTCTTCAATCTGAATTGGATGCCAAACGAACTAAACTATCAACACTTGAAGAGTTGAGTGATAAGATTGATGAGTTAGACGCTGAAATTGCAAAACTTGAAAAAAATGTAGAATATTTCAAAAAAACCGATGCTGAGCAAACTGAACAATACCTTGCTGCAGCTGAAAAAGACTTAGCTGATAAAAAAGCTGAATTGGAGAAAACTGAAGCTGACCTTAAGAAAGCAGTTAATGAGCCAGAAAAACCAGCTGAAGAAACTCCAGCTCCAGCACCAAAACCAGAAAAAACAGATGATCAACAAGCTGAAGAAGACTATGCTCGTAGATCAGAAGAAGAATATAACCGCTTGCCCCAACAGCAACCGCCAAAAGCAGAAAAACCAGCTCCAGCACCAAAACCAGAGCAACCAGTTCCT
**Patient 1 and Patient 2 PspA Protein 100% Alignment**

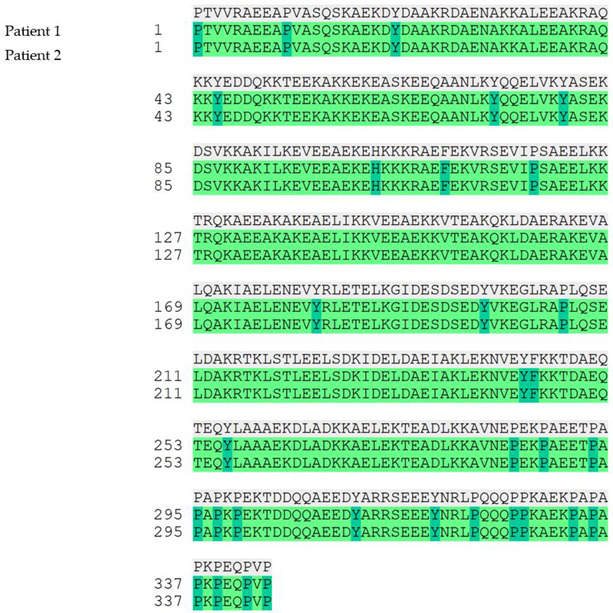

Identical residues are in black on a green background.
